# AI for IMPACTS Framework for Evaluating the Long-Term Real-World Impacts of AI-Powered Clinician Tools: Systematic Review and Narrative Synthesis

**DOI:** 10.2196/67485

**Published:** 2025-02-05

**Authors:** Christine Jacob, Noé Brasier, Emanuele Laurenzi, Sabina Heuss, Stavroula-Georgia Mougiakakou, Arzu Cöltekin, Marc K Peter

**Affiliations:** 1 FHNW, University of Applied Sciences and Arts Northwestern Switzerland Windisch Switzerland; 2 Institute of Translational Medicine, Department of Health Science and Technology, ETH Zurich Zurich Switzerland; 3 ARTORG Center for Biomedical Engineering Research, University of Bern Bern Switzerland; 4 University of Nicosia Nicosia Cyprus

**Keywords:** eHealth, assessment, adoption, implementation, artificial intelligence, clinician, efficiency, health technology assessment, clinical practice

## Abstract

**Background:**

Artificial intelligence (AI) has the potential to revolutionize health care by enhancing both clinical outcomes and operational efficiency. However, its clinical adoption has been slower than anticipated, largely due to the absence of comprehensive evaluation frameworks. Existing frameworks remain insufficient and tend to emphasize technical metrics such as accuracy and validation, while overlooking critical real-world factors such as clinical impact, integration, and economic sustainability. This narrow focus prevents AI tools from being effectively implemented, limiting their broader impact and long-term viability in clinical practice.

**Objective:**

This study aimed to create a framework for assessing AI in health care, extending beyond technical metrics to incorporate social and organizational dimensions. The framework was developed by systematically reviewing, analyzing, and synthesizing the evaluation criteria necessary for successful implementation, focusing on the long-term real-world impact of AI in clinical practice.

**Methods:**

A search was performed in July 2024 across the PubMed, Cochrane, Scopus, and IEEE Xplore databases to identify relevant studies published in English between January 2019 and mid-July 2024, yielding 3528 results, among which 44 studies met the inclusion criteria. The systematic review followed PRISMA (Preferred Reporting Items for Systematic reviews and Meta-Analyses) guidelines and the Cochrane Handbook for Systematic Reviews. Data were analyzed using NVivo through thematic analysis and narrative synthesis to identify key emergent themes in the studies.

**Results:**

By synthesizing the included studies, we developed a framework that goes beyond the traditional focus on technical metrics or study-level methodologies. It integrates clinical context and real-world implementation factors, offering a more comprehensive approach to evaluating AI tools. With our focus on assessing the long-term real-world impact of AI technologies in health care, we named the framework AI for IMPACTS. The criteria are organized into seven key clusters, each corresponding to a letter in the acronym: (1) I—integration, interoperability, and workflow; (2) M—monitoring, governance, and accountability; (3) P—performance and quality metrics; (4) A—acceptability, trust, and training; (5) C—cost and economic evaluation; (6) T—technological safety and transparency; and (7) S—scalability and impact. These are further broken down into 28 specific subcriteria.

**Conclusions:**

The AI for IMPACTS framework offers a holistic approach to evaluate the long-term real-world impact of AI tools in the heterogeneous and challenging health care context and lays the groundwork for further validation through expert consensus and testing of the framework in real-world health care settings. It is important to emphasize that multidisciplinary expertise is essential for assessment, yet many assessors lack the necessary training. In addition, traditional evaluation methods struggle to keep pace with AI’s rapid development. To ensure successful AI integration, flexible, fast-tracked assessment processes and proper assessor training are needed to maintain rigorous standards while adapting to AI’s dynamic evolution.

**Trial Registration:**

reviewregistry1859; https://tinyurl.com/ysn2d7sh

## Introduction

### Background

Artificial intelligence (AI) is profoundly transforming health care across a range of applications, enhancing both clinical outcomes and operational efficiency. In medical imaging, AI algorithms improve diagnostic accuracy by analyzing complex imaging data, such as from magnetic resonance imaging and computed tomography scans, for highly precise and rapid clinical diagnostics [1]. Decision support systems powered by AI assist clinicians in making evidence-based decisions by providing real-time data-driven insights and predictive analytics [2]. Large language models are increasingly used for generating detailed medical reports and streamlining triage processes by analyzing and summarizing patient data quickly and accurately [3]. In addition, innovative digital health technologies such as electronic skins use wearable sensor technologies and AI to offer continuous, real-time monitoring of various health indicators, further enhancing personalized care [4]. These advancements have the potential to contribute to a more efficient, accurate, responsive, and holistic health care, reshaping how patient care is delivered and managed.

Despite the growing body of literature on AI in health care, its implementation has lagged behind other industries [5,6]. Previous studies have highlighted substantial barriers to the successful adoption of AI in health care, including issues related to trust; potential risks of harm; accuracy and perceived usefulness; reproducibility; evidentiary standards; and ethical, legal, and societal concerns [7,8]. In addition, uncertainty surrounding postadoption outcomes further complicates the implementation process [7].

A significant barrier identified by health care leaders worldwide is that despite the emergence of various new frameworks for assessing AI in health care, most focus primarily on the quality of study methodologies or technical aspects [9,10]. There remains a lack of a comprehensive, systematic framework that assesses the real-world impact of AI and offers guidance on clinical implementation, monitoring, procurement, and evaluation [9,11]. Most research overlooks the complex, multistep process required for successful AI integration, leaving critical gaps in understanding how to effectively implement and sustain AI tools in clinical practice [9,11]. As a result, the adoption of AI in clinical practice has fallen short of expectations, with only a few algorithms showing sustained clinical impact [12]. This gap is often due to inadequate or incomplete evaluation and the lack of universally recognized standards for AI assessment. The limited understanding of AI’s true added value in health care highlights the need for a more comprehensive evaluation framework [13-15]. To ensure confidence in the added clinical value and successful integration of AI into health care workflows, a practical, comprehensive tool is needed so that the translational readiness of AI systems can be evaluated. Current approaches assessing AI in health care often focus on foundational technical metrics such as sensitivity and specificity, which fail to capture the full clinical impact [13,16]. A robust valuation should encompass factors such as patient outcomes, effects on clinical decision-making, workflow efficiency, and the tangible benefits for patients to fully determine AI’s true contribution to and impact on health care [10,17,18].

In the context outlined earlier, regulatory approval is an important milestone for demonstrating overall performance, although the scientific evidence supporting AI tools in health care remains limited compared to traditional medical standards [9,19]. In addition, new regulations are being introduced to keep pace with rapidly evolving AI technologies, such as the European Union (EU) AI Act, which aims to ensure the trustworthiness of high-risk AI tools including those used in health care [20]. Despite the potentially positive impact of regulatory frameworks on AI-related developments, a recent study revealed that nearly half of Food and Drug Administration (FDA)–authorized AI devices lacked clinical validation data, raising concerns about their safety and effectiveness [21]. Without robust clinical validation, these technologies could pose significant risks to patient care. Despite efforts to create reporting guidelines for AI in health care, such as Standard Protocol Items Recommendations for Interventional Trials–Artificial Intelligence (SPIRIT-AI) [13], CONSORT-AI (Consolidated Standards of Reporting Trials–Artificial Intelligence) [14], Standards for Reporting of Diagnostic Accuracy Studies–Artificial Intelligence [22], Checklist for Artificial Intelligence in Medical Imaging [23], Prediction Model Risk of Bias Assessment Tool–Artificial Intelligence [24], and others, a unified international consensus on the evaluation of AI-based tools has yet to be established. While these guidelines address key methodological issues and share significant overlap, indicating the importance of certain assessment criteria, the absence of a standardized, universally accepted framework remains a significant challenge [4]. This lack of consensus complicates the consistent evaluation and implementation of AI technologies in clinical practice.

### Objectives

The goal of this study was to develop a comprehensive framework for assessing the impact of AI tools in health care. This involved synthesizing and consolidating the various evaluation criteria found in existing literature regarding the quality and impact of AI tools. On the basis of the outcomes of this study, we plan on validating the framework through expert consensus using the Delphi process. However, this validation effort will be addressed in the subsequent phase of the project and is beyond the scope of this foundational paper. This approach aims to create a rigorous, evidence-based structure for AI evaluation, ensuring its relevance and applicability in health care settings.

In doing so, we adopted the perspective of the World Health Organization (WHO) on AI in health care, defining it as “the ability of algorithms and software to analyze complex medical data and support health care providers by improving decision-making, predicting outcomes, and enhancing clinical efficiency” [25]. AI tools in health care span a broad spectrum of applications, such as (1) diagnostic support, (2) prognosis of diseases course, (3) personalized treatment recommendations, (4) patient monitoring, and (5) overall health management, driving innovation across the health care landscape [25].

To address this, a systematic review was conducted to offer a comprehensive and current analysis of the criteria used in existing research to evaluate the quality and impact of AI in health care, from technological, social, and organizational perspectives. The review also explores the potential implications of AI implementation for key stakeholders and offers recommendations on how to effectively assess AI-powered clinical tools under consideration for clinical impact. This study builds upon and extends the findings of a prior research project, which examined the sociotechnical assessment criteria for patient-facing eHealth tools, that is already published [26,27].

We believe the results of this review will provide valuable insights for clinicians, pharmaceutical leaders, insurance professionals, technology providers, and policy makers by presenting an up-to-date, thorough overview of the criteria used to assess AI-powered clinical tools. These insights will help stakeholders make informed decisions about which tools to implement, recommend to patients, invest in, partner with, or provide reimbursement for, based on their assessed quality and potential impact.

## Methods

### Overview

The methodology for this review was based on established best practices, specifically following the PRISMA (Preferred Reporting Items for Systematic reviews and Meta-Analyses) guidelines [28] and the Cochrane Handbook for Systematic Reviews of Interventions [29]. These frameworks were chosen to ensure a rigorous and methodologically sound approach to the systematic literature review process. All review methods were predetermined and documented in advance, with the protocol being publicly registered in the research registry (reviewregistry1859) to enhance transparency and accountability [30]. The primary research question guiding this systematic review was the following: What technical, social, and organizational criteria should be considered when assessing the quality and impact of AI-powered clinical tools? This question served as the foundation for the analysis and exploration of the criteria relevant to AI’s evaluation in clinical settings. The study remained highly consistent with the initial protocol from a methodological standpoint, adhering to the predefined review question; search strategy; databases; inclusion and exclusion criteria; participants, intervention, comparators, and outcomes (PICO) framework elements; data extraction strategy; quality assessment; and data synthesis approach as originally outlined. The only variation from the protocol was in the presentation of the findings: rather than merely listing the results as an inventory of criteria, we organized them into a cohesive framework. This structured approach enhances both the memorability and practical applicability of the results in real-world settings.

### Search Strategy

A comprehensive search of the PubMed, Cochrane, Scopus, and IEEE Xplore databases was conducted in July 2024 to identify relevant studies. The review was limited to peer-reviewed papers published in English between January 2019 and mid-July 2024. We focused on this specific time frame and limited the search to the last 5 years to ensure the findings reflect the most recent advancements and challenges, particularly with the emergence of new generative AI technologies. Going back further would have added limited value, as older studies may not capture the rapid technological shifts and evolving complexities that are relevant today. Only fully published research articles were included, while other formats, such as editorials and study protocols, were excluded from the analysis. In accordance with the Cochrane Handbook for Systematic Reviews of Interventions, we chose not to include articles sourced through manual reference list searches, as “positive studies are more likely to be cited,” which could introduce bias [29].

This systematic review focused on AI-powered tools designed specifically for clinicians, excluding tools meant solely for patients or medical students as these will most likely not reflect the implementation aspects in real-world health care organizations. The search strategy targeted manuscripts with titles including the terms “AI” or “Artificial Intelligence,” reflecting the intervention focus on AI technologies. Outcomes of interest were assessment criteria, captured through titles containing the terms “assessment,” “assess,” “evaluation,” “evaluating,” “effectiveness,” “efficacy,” “quality,” “efficiency,” “usability,” or “usefulness,” as well as abstracts mentioning “criteria,” “framework,” “method,” “methodology,” “methodologies,” “measurement,” “toolkit,” “tool,” “tools,” “approach,” or “scorecard.” No condition-based restrictions were applied, aligning with a broad approach to capture all relevant studies on assessment methodologies for clinician-targeted AI tools.

Textbox 1 illustrates the search string designed using the PICO framework. To ensure the relevance of the retrieved papers, the search was mostly restricted to manuscript titles, focusing on studies that addressed AI assessment criteria comprehensively rather than those evaluating specific tools or pilot studies. Because comparators were not relevant to this review, they were excluded from the search parameters.

**The search string according to the participants, intervention, comparators, and outcomes framework.**

**Participants: clinicians**

- Focus on artificial intelligence (AI)–powered tools for clinicians, excluding those designed solely for patients or medical students.

**Intervention: AI-powered clinician tools**

- Focus is on AI-powered clinician tools: the search targeted manuscript titles containing the terms (AI OR “Artificial Intelligence”).

**Comparator: not applicable**

- There were no restrictions on eligible conditions for inclusion.

**Outcome: assessment criteria**

- The search targeted manuscript titles also containing AND (assessment OR assess OR evaluation OR evaluating OR effectiveness OR efficacy OR quality OR efficiency OR usability OR usefulness). As well as manuscript tiles and abstracts containing AND (criteria OR framework OR method OR methodology OR methodologies OR measurement OR toolkit OR tool OR tools OR approach OR scorecard).

### Study Selection

Two researchers (CJ and EL) participated in the screening, eligibility, and inclusion phases of the study. Any discrepancies during these stages were resolved through discussion among them. If consensus could not be reached, a third coauthor was consulted to make the final decision. The team used the open-source Rayyan app (Qatar Computing Research Institute) to streamline collaborative screening efforts [31]. The screening process took place between July and August 2024.

The inclusion and exclusion criteria, outlined in Textbox 2, were developed following the PICO framework. Included studies centered on AI-powered tools in clinical settings, addressing criteria to assess the quality and impact of these tools. Eligible studies were peer-reviewed, published between January 2019 and mid-July 2024, and written in English. Exclusions were made for studies involving only patients or medical students as they were not likely to reflect implementation factors, AI technologies outside clinical settings (eg, patient use chatbots), studies assessing specific tools in isolation, or frameworks solely evaluating AI research methodology or clinical trials rather than the implementation of the tools in real-world settings. Editorials, study protocols, and non-English publications were also excluded.

Following the completion of the screening process and resolution of any conflicting views among the researchers, CJ and EL proceeded to assess the full texts of the selected studies for eligibility. Any remaining disagreements were addressed through consultation with a third coauthor. CJ evaluated the risk of bias using the Critical Appraisal Skills Program (CASP) checklist [32], which assesses key quality criteria in the included studies. These criteria include the following: the presence of a clear statement of the research aims, the appropriateness of the methodology for the research objectives, the suitability of the research design in addressing those aims, the relevance of the recruitment strategy, the adequacy of data collection methods in relation to the research question, the consideration given to the researchers’ roles, the evaluation of ethical issues, the rigor of data analysis, the clarity of the study’s findings, and whether the researchers discussed the study’s contribution to existing knowledge, such as its implications for current practice, policy, or relevant literature. The results of this appraisal are available in Multimedia Appendix 1.

**Inclusion and exclusion criteria according to the participants, intervention, comparators, and outcomes framework.**

**Inclusion criteria**

- Participants: focused on clinicians
- Intervention: focused on artificial intelligence (AI)–powered clinician tools
- Comparators: does not apply
- Outcomes: addresses the different criteria used to assess the quality and impact of AI-powered clinician tools regardless of the condition
- Publication type: peer-reviewed and published papers
- Time frame: studies published between January 2019 and mid-July 2024
- Language: studies published in English

**Exclusion criteria**

- Participants: focused solely on patients or medical students
- Intervention: technologies used outside of clinical environments, such as chatbots used by patients to obtain health care information
- Comparators: does not apply
- Outcomes: individual assessments of pilot studies singling out specific tools, and assessment frameworks that focus on the reporting and methodological quality of AI research and clinical studies rather than evaluating the AI tool itself
- Publication type: editorials and study protocols
- Time frame: studies published before January 2019 or after mid-July 2024
- Language: studies published in languages other than English

### Data Collection and Synthesis

The procedures and outcomes across the included studies were too diverse to support a quantitative analysis. As a result, a narrative synthesis was used following the sociotechnical approach, organized around the social, organizational, and technical criteria used to evaluate the quality and impact of AI-powered tools for clinicians. The authors were influenced by the sociotechnical theory, which emphasizes that the design and performance of innovations can only be fully understood when both social and technical aspects are considered as interdependent components of a larger system [33]. This approach aligns with recommendations from several scholars who advocate for moving beyond purely technology-focused frameworks to incorporate the broader context, including societal and implementation factors [34-36]. To facilitate this process, NVivo (version 1.7.2; Lumivero), a qualitative data analysis software, was used.

Data coding began with a preliminary extraction grid, which was structured around themes derived from previous research and established technology acceptance frameworks. The initial codebook was informed by our prior work on factors influencing eHealth evaluation and adoption [26,27,36-38], with additional codes being incorporated as new themes emerged during the review. Thematic analysis, as outlined by Braun and Clarke [39], was conducted to identify and extract themes based on the social, technical, and organizational assessment criteria relevant to the research question. This analysis followed 7 key phases: familiarizing with the data, generating initial codes, searching for themes, reviewing themes, defining and naming themes, linking themes to explanatory frameworks, and producing the final report.

In line with the approach of Braun and Clarke [40], we opted not to use interrater reliability as it aligns more closely with quantitative methods and standardized interpretation. Thematic analysis in a qualitative context prioritizes depth, subjectivity, and the unique insights each researcher brings to the data. Rather than using numerical reliability measures such as interrater reliability, reliability in this approach is often ensured through collaborative discussions that allow for consensus and a nuanced understanding of the themes. Accordingly, the first author, CJ, conducted the initial analysis and coding and NB reviewed the coding. Any cases of disagreement were discussed and mutually agreed upon in conjunction with a third author. Using the sociotechnical framework as our guide, we developed our initial codebook and grouped the criteria accordingly. This approach ensures a holistic evaluation of each tool, capturing the complex interdependencies between technical capabilities, social contexts, and organizational fit and readiness. By doing so, we moved beyond a narrow technical focus or methodological evaluation at the study level, ensuring that the social and organizational dimensions are fully integrated into the analysis. As a result, this work prioritizes the often-overlooked social and organizational dimensions that are critical for the successful implementation of AI technologies. Unlike frameworks that focus solely on clinical study quality, our analysis and synthesis specifically emphasize social and organizational factors such as user trust, support and training, interoperability, and integration.

However, we intentionally did not apply any hierarchy or prioritization within this foundational framework, as the purpose here is to treat all criteria as equally significant. Prioritization and potential gap identification will occur in the next phase (beyond the scope of this paper), where the Delphi process will engage an expert panel to further refine and prioritize these criteria. The coding and analysis process was carried out from August to October 2024.

## Results

### Study Selection Flow and Characteristics of the Included Studies

**Figure 1:**
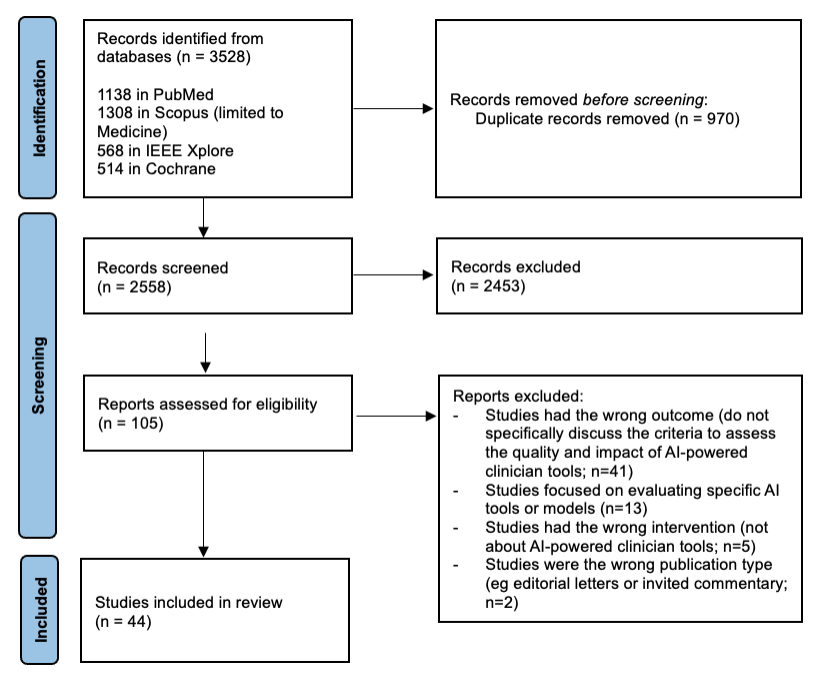
Study selection flow diagram based on the PRISMA (Preferred Reporting Items for Systematic Reviews and Meta-Analyses) guidelines. AI: artificial intelligence.

Figure 1 presents the PRISMA flow diagram, illustrating the progression of study selection during the systematic review. It details the number of records identified, screened, included, and excluded, along with reasons for exclusion. After applying these criteria, 44 articles were selected for the qualitative synthesis.

Table 1 outlines the characteristics of these studies, offering insights into their research methodologies, geographic distributions, and clinical focuses. This comprehensive overview highlights the diversity of approaches and topics addressed within the included studies.

**Table 1:** Characteristics of the included studies (N=44).

Study characteristics				Studies, n (%)		References
**Country of authors**						
	Multiple		21 (48)		[41-61]	
	United States		5 (11)		[62-66]	
	France		3 (7)		[67-69]	
	Netherlands		3 (7)		[70-72]	
	Australia		2 (5)		[73,74]	
	Canada		2 (5)		[75,76]	
	**Others**					
		China	1 (2)		[77]	
		Denmark	1 (2)		[78]	
		Germany	1 (2)		[79]	
		Greece	1 (2)		[80]	
		India	1 (2)		[81]	
		Saudi Arabia	1 (2)		[82]	
		Sweden	1 (2)		[83]	
		United Kingdom	1 (2)		[84]	
**Focus (some papers encompassed multiple areas of focus)**						
	No specific focus		9 (21)		[49,54,56,63,65,67,68,70,75]	
	**Clinical focus**					
		Cardiovascular	3 (7)		[43,58,59]	
		Dermatology	2 (5)		[42,66]	
		ENT^a^	1 (2)		[82]	
		Medical imaging	12 (27)		[41,45,46,55,64,69,74,76,78,81,83,84]	
		Nuclear medicine	1 (2)		[48]	
		Radiation oncology	1 (2)		[47]	
	**Technology focus**					
		ANN^b^	2 (5)		[48]	
		CDSSs^c^	3 (7)		[52,53,77]	
		DQMs^d^	1 (2)		[51]	
		LLMs^e^	3 (7)		[57,62,73]	
		ML^f^	2 (5)		[46,53]	
		Prediction models	2 (5)		[58,71]	
	**Thematic focus**					
		EEs^g^	4 (9)		[44,61,72,79]	
		Ethics and equity	3 (7)		[60,63,66]	
		Explainability	1 (2)		[80]	
		Regulatory and trust	2 (5)		[50,70]	
**Paper type**						
	**Original research**					
		Delphi process	3 (7)		[44,75,77]	
		Survey or questionnaire	2 (5)		[80,82]	
	Expert consensus		3 (7)		[42,57,69]	
	Expert perspective or comment		9 (21)		[51,52,54,55,62,64-66,76]	
	Guidelines or statements		6 (14)		[41,47,48,58,60,63]	
	Policy brief		1 (2)		[70]	
	Review		10 (23)		[45,46,50,53,56,59,74,81,83,84]	
	Scoping review		6 (14)		[43,68,71,73,78,79]	
	Systematic review		4 (9)		[49,61,67,72]	
**Publication year**						
	2019 (from January)		2 (5)		[46,52]	
	2020		2 (5)		[53,60]	
	2021		5 (11)		[50,55,56,70,74,77]	
	2022		10 (23)		[42,45,48,59,71,72,78,79,83]	
	2023		12 (27)		[43,47,51,58,61,63,65,67,69,73,75,76,80,81]	
	2024 (until mid-July)		13 (30)		[41,44,49,54,57,62,64,66,68,82,84]	
**Frameworks resulting from the included studies**						
	ABCDS^h^		1 (2)		[63]	
	CHEERS-AI^i^		1 (2)		[44]	
	CLEAR^j^		1 (2)		[42]	
	DQM^k^		1 (2)		[51]	
	DRIM France AI grid^l^		1 (2)		[69]	
	ECLAIR^m^		1 (2)		[55]	
	HEAL^n^		1 (2)		[66]	
	MAS-AI^o^		1 (2)		[78]	
	RADAR^p^		1 (2)		[41]	
	RELAINCE guidelines^q^		1 (2)		[48]	
	R‑AI‑DIOLOGY checklist^r^		1 (2)		[45]	
	TEHAI^s^		1 (2)		[56]	
	TREE^t^		1 (2)		[60]	
**Frameworks used in or referred to in the included studies**						
	CHEERS^u^		3 (7)		[44,61,72]	
	CLAIM^v^		4 (9)		[67,76,78,81]	
	CONSORT-AI^w^		6 (14)		[42,44,61,67,68,74]	
	DECIDE-AI^x^		2 (5)		[42,84]	
	FUTURE-AI^y^		1 (2)		[41]	
	GEP-HI^z^		1 (2)		[52]	
	HTA^aa^		6 (14)		[43,59,61,67,68,78]	
	MAST^ab^		2 (5)		[43,78]	
	PROBAST-AI^ac^		4 (9)		[42,44,67,74]	
	QAMAI^ad^		1 (2)		[57]	
	QMS^ae^		1 (2)		[65]	
	RQS^af^		1 (2)		[76]	
	SPIRIT-AI^ag^		6 (14)		[42,44,61,67,68,74]	
	STARD-AI^ah^		3 (7)		[42,67,74]	
	STARE-HI^ai^		1 (2)		[52]	
	TRIPOD-AI^aj^		7 (16)		[42,44,58,60,63,68,74]	

^a^ENT: ear, nose, and throat.

^b^ANN: artificial neural network.

^c^CDSS: clinical decision support system.

^d^DQM: diagnostic quality model.

^e^LLM: large language model.

^f^ML: machine learning.

^g^EE: economic evaluation.

^h^ABCDS: Algorithm-Based Clinical Decision Support.

^i^CHEERS-AI: Consolidated Health Economic Evaluation Reporting Standards for Interventions That Use Artiﬁcial Intelligence.

^j^CLEAR: Derm Consensus Guidelines from the International Skin Imaging Collaboration Artificial Intelligence Working Group.

^k^DQM: Diagnostic Quality Model.

^l^DRIM France AI grid: French community grid for the evaluation of radiological artificial intelligence solutions.

^m^ECLAIR: Evaluating Commercial Artificial Intelligence Solutions in Radiology.

^n^HEAL: Health Equity Assessment of Machine Learning Performance.

^o^MAS-AI: Model for Assessing the Value of Artificial Intelligence in Medical Imaging.

^p^RADAR: Radiology Artificial Intelligence Deployment and Assessment Rubric.

^q^RELAINCE guidelines: Recommendations for Evaluation of Artificial Intelligence for Nuclear Medicine.

^r^R‑AI‑DIOLOGY checklist: a practical checklist for evaluation of artificial intelligence tools in clinical neuroradiology.

^s^TEHAI: Translational Evaluation of Healthcare Artificial Intelligence.

^t^TREE: transparency, reproducibility, ethics, and effectiveness.

^u^CHEERS: Consolidated Health Economic Evaluation Reporting Standards.

^v^CLAIM: Checklist for Artificial Intelligence in Medical Imaging.

^w^CONSORT-AI: Consolidated Standards of Reporting Trials–Artificial Intelligence.

^x^DECIDE-AI: Reporting Guideline for the Developmental and Exploratory Clinical Investigations of Decision Support Systems Driven by Artificial Intelligence.

^y^FUTURE-AI: International consensus guideline for trustworthy and deployable artificial intelligence in health care.

^z^GEP-HI: Good Evaluation Practice in Health Informatics.

^aa^HTA: Health Technology Assessment.

^ab^MAST: Model for Assessment of Telemedicine.

^ac^PROBAST-AI: Prediction Model Risk of Bias Assessment Tool–Artiﬁcial Intelligence.

^ad^QAMAI: Quality Analysis of Medical Artificial Intelligence.

^ae^QMS: Quality Management System.

^af^RQS: Radiomics Quality Score.

^ag^SPIRIT-AI: Standard Protocol Items: Recommendations for Interventional Trials–Artiﬁcial Intelligence.

^ah^STARD-AI: Standards for Reporting of Diagnostic Accuracy Studies–Artiﬁcial Intelligence.

^ai^STARE-HI: Statement on Reporting of Evaluation Studies in Health Informatics.

^aj^TRIPOD-AI: Transparent Reporting of a Multivariable Prediction Model for Individual Prognosis or Diagnosis–Artiﬁcial Intelligence.

### Critical Appraisal

We evaluated the quality of the included studies using the CASP checklist [32]. This tool was selected due to the variety of methodologies used in the studies and the narrative approach of our synthesis, which differed from meta-analyses and other quantitative methods. The CASP is widely recognized as the most frequently used tool for appraising the quality of qualitative evidence in health research, with endorsement from the Cochrane Qualitative and Implementation Methods Group [85]. The studies included in our review used a range of methodologies (quantitative, qualitative, mixed methods, and systematic literature reviews), which meant that some questions on the checklist were not applicable to all study types. As per the checklist’s recommendations, we did not assign scores to the studies.

Following the critical appraisal of the 44 studies, several issues were identified. While all studies clearly stated their aims, presented well-defined findings, and provided valuable insights for health care stakeholders, 21 (N=44, 48%) studies lacked a dedicated methods section, making it difficult to assess the appropriateness and suitability of their approach. Similarly, the absence of clear methods in these studies hindered the evaluation of the research design and data collection techniques.

In addition, out of 44 studies, 25 (57%) studies did not detail their analysis methods, making it challenging to gauge the rigor and reliability of their approach. Furthermore, 28 (64%) studies lacked validation of their findings, while 8 (18%) offered only partial validation (eg, expert consensus), highlighting the need for empirical validation in real-world clinical applications to ensure the findings’ robustness. The comprehensive quality assessment of the included studies can be found in Multimedia Appendix 1.

Studies were not excluded based on the results of the quality assessment, as this was unlikely to significantly impact the definition of the assessment criteria or the development of the aggregated framework. However, the quality assessment offered valuable insight into the overall robustness of the development processes behind the existing frameworks, helping to gauge the strength and reliability of the evidence presented [85]. An in-depth exploration of this topic can be found in the Discussion section, where the challenges associated with current initiatives and frameworks are examined.

### Synthesized Assessment Criteria

We synthesized comparable measures from various papers, frameworks, and initiatives, ultimately identifying a set of unique criteria that reflected all relevant assessment methods referenced in the included studies. Notably, several criteria are closely interrelated and could fit into multiple categories; however, they were placed in the most appropriate category based on their significance and impact. For instance, while “user trust” and “model explainability” are inherently linked, because trust often correlates with the level of explainability provided by an AI system, we categorized trust under the cluster “acceptability, trust, and training,” which focuses on user-centric aspects, whereas “explainability” was assigned to the cluster evaluating model performance metrics, given its technical focus. In addition, we intentionally included assessment criteria applicable to high-risk tools, enabling us to compile a more comprehensive list. We recognized that not all criteria would apply to lower-risk AI-powered health care tools, such as patient safety assessments, which are more relevant to high-risk tools that pose potential safety concerns. We are guided by National Institute for Health and Care Excellence’s Evidence Standards Framework for Digital Health Technologies to assess and understand the risk levels of health care technologies [86].

**Figure 2:**
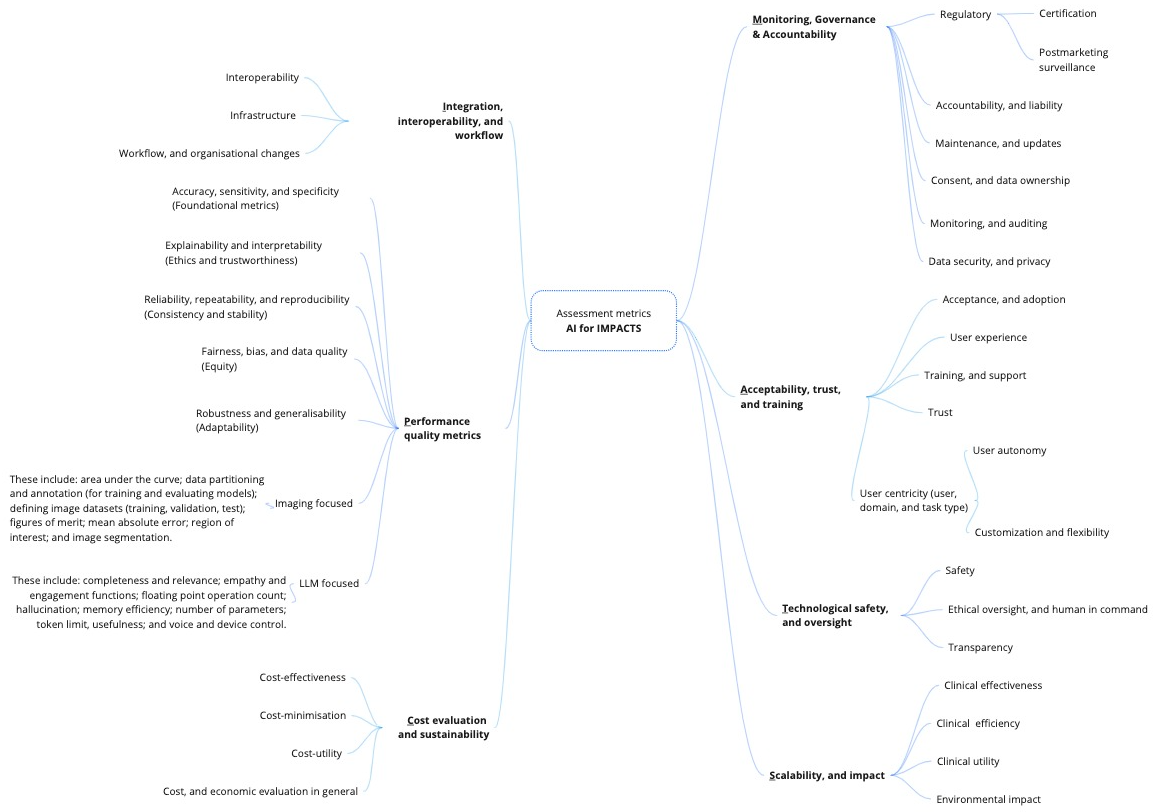
Visual overview of the aggregated assessment criteria, organized into clusters and subcriteria. AI: artificial intelligence.

Figure 2 provides a visual overview of the aggregated criteria, organized into clusters and subclusters, while Table 2 presents these criteria grouped into 7 primary clusters and their respective subcriteria, outlining their occurrences across the included studies, along with their definitions and corresponding references. A detailed exploration of each criteria cluster and its corresponding subcriteria is provided in the Discussion section.

**Table 2:** Assessment criteria, their definitions, occurrence, and respective references (N=44).

Criteria			Definition		Studies, n (%)		Studies in which the criteria occurred
**Integration**							
	Infrastructure	The underlying technological, hardware, and software systems required to support the deployment and scalability of the AI^a^ tool		15 (34)		[41,44,49,53-55,60,65,68,69,71,75,76,78,84]	
	Interoperability	The AI tool’s ability to seamlessly exchange and integrate data with different health care platforms and devices		19 (43)		[41,45,51,53-56,59,64,67-69,71,74-78,82]	
	Workflow and organizational changes	The degree to which the AI tool impacts existing clinical workflows and health care operations, ensuring minimal disruption while enhancing efficiency, communication, and overall care delivery		22 (50)		[41,45,47,49,51-56,58,64,65,67-69,71,76,78,79,82,84]	
**Monitoring, governance, and accountability**							
	Accountability and liability	The clear attribution of responsibility for errors or outcomes and the establishment of legal and ethical frameworks to address potential issues and ensure proper recourse		13 (30)		[49-51,54,55,63-65,70,75,78,81,84]	
	Consent and data ownership	Evaluates the processes for obtaining informed consent from patients regarding the use of their data and ensuring clear policies on data ownership, privacy, and control		5 (11)		[49,54,68,78,81]	
	Maintenance and updates	Evaluates the processes for ongoing support, including regular updates and bug fixes, to ensure the AI tool remains effective, secure, and aligned with evolving medical standards and practices		13 (30)		[44,45,49,50,52,53,55,59,65,69,71,76,77]	
	Monitoring and governance	Evaluates the systems in place for overseeing the AI’s performance, including regular assessments and audits to ensure ethical use and effectiveness		22 (50)		[44,48-56,59,60,64,65,68,69,71,74,76,81,83,84]	
	Regulatory compliance	Evaluates adherence to established regulations throughout the AI tool’s life cycle, including ongoing monitoring and reporting after deployment to ensure continued safety, efficacy, and adherence to legal requirements		23 (52)		[45,49-55,59,60,63-65,67-69,72,74,76,78,81,83,84]	
	Security and privacy	Evaluates the measures implemented to protect sensitive patient data from unauthorized access and breaches while ensuring compliance with privacy regulations		26 (59)		[45,51-56,60,62,64-71,73-75,77,78,80-83]	
**Performance quality metrics**							
	Accuracy, sensitivity, and specificity (foundational metrics)	Accuracy: the proportion of correct predictions (both true positives and true negatives) out of all predictions. It gives an overall measure of performance but may be misleading if the dataset is imbalanced (ie, when one class dominates). Sensitivity (recall): the ability of the model to correctly identify true positives (ie, people with the condition). In health care, this often refers to how well the model detects cases like diseases. High sensitivity ensures that most cases of the disease are caught, reducing the chance of missing sick patients. Specificity: the ability to correctly identify true negatives (ie, people without the condition). High specificity means the model avoids false positives, reducing unnecessary interventions for healthy people		26 (59)		[41,43,45-53,57,58,60,62,67,69,70,73,74,76,80-84]	
	Explainability and interpretability (ethics and trustworthiness)	Explainability: refers to the degree to which the model’s predictions and decisions can be understood by humans. In health care, explainability is crucial because clinicians need to trust AI recommendations and understand why the AI made a particular decision. Interpretability: closely related to explainability, it is about how easily a human can comprehend the internal workings of the model. For example, an interpretable model may allow clinicians to track how specific features (like patient age or laboratory results) influenced the AI’s prediction		19 (43)		[45,47-49,51,52,55,60,62,67,68,71,73-75,78,80,81,83]	
	Fairness (equity)	Fairness: ensures that the AI model does not systematically discriminate against any specific group of people (eg, based on race, gender, or socioeconomic status). Fairness in health care is key to avoid bias in diagnoses or treatments.		32 (73)		[42-47,49,51-56,58,60,62-69,71,73-75,77,78,81,83,84]	
	Reliability, repeatability, and reproducibility (consistency and stability)	Reliability: refers to the consistency of the model over time. Can the AI be trusted to perform in the same way under similar conditions in the future? Repeatability: the ability of the model to provide consistent results when the same input is given multiple times in the same environment. In health care, this ensures that if a patient is reevaluated using the same AI tool, it will give the same outcome. Reproducibility: refers to how well the model performs when applied to different datasets or by different teams. This is critical in health care, where models trained on one population must still perform well when tested on different populations or data collected in different hospitals.		24 (55)		[42,43,45,48,50-56,58-60,64,67,68,71,74,76,77,81,83,84]	
	Robustness and generalizability (adaptability)	Robustness: the model’s ability to maintain performance despite slight variations or noise in the input data. In a health care setting, this might mean the model works well even with slightly lower-quality images or laboratory results from different equipment. Generalizability: the ability of the model to perform well on new, unseen data that may differ from the training data. In health care, it is crucial that an AI model trained in one hospital or region can generalize to others.		23 (52)		[41-46,48,49,52,54-56,58,60,64,67,68,70,71,75,76,83,84]	
	Imaging-focused	These may include area under the curve; data partitioning and annotation (for training and evaluating models); defining image datasets (training, validation, and testing); figures of merit; mean absolute error; region of interest and image segmentation		10 (23)		[42,45-48,55,69,76,81,83]	
	Large language model-focused	These may include completeness and relevance; empathy and engagement functions; ﬂoating point operation count; hallucination; memory efficiency; number of parameters; token limit and usefulness; voice and device control		3 (7)		[57,62,73]	
**Acceptability, trust, and training**							
	Acceptance and adoption	Evaluates how well the AI tool is embraced by health care professionals and patients, including their willingness to integrate it into routine practice		18 (41)		[47,49,52-54,56,60,63,65,67-69,72,73,75,77,78,82]	
	Training and support	Evaluates the effectiveness and availability of resources provided to users for learning and using the AI tool, ensuring they have the necessary guidance and assistance for successful implementation and operation		17 (39)		[45,49,52-55,64,65,67,69,71,76,78,80-82,84]	
	Trust	Evaluates the degree to which health care professionals and patients believe in the reliability, accuracy, and ethical considerations of the AI tool, influencing their willingness to use it		11 (25)		[45,47,49,52,60,62,68,73,75,82,84]	
	Usability	Evaluates how easily and effectively health care professionals and patients can interact with and use the AI tool, ensuring it enhances rather than hinders the user experience and clinical workflows		18 (41)		[47,49-53,55,56,62,63,68,71,73,75-77,82,84]	
	User centricity (user, domain, and task type)	Evaluates how well the AI tool is designed to meet the specific needs, preferences, and contexts of its users, domain-specific requirements, and task types it is intended to support		19 (43)		[42,45,49,52,53,55,56,58,60,62,64,65,71,73,75-77]	
**Cost and economic evaluation**							
	Costs and economic evaluation in general	Evaluates the financial implications of implementing the AI tool, ensuring it provides value without imposing excessive financial burdens on health care systems or patients		18 (41)		[44,49,51,55,59-61,64,67-69,72,73,75,76,79,82,84]	
	Cost-effectiveness analysis	Compares the relative costs and outcomes of different interventions. The outcomes are typically measured in natural units like life years saved, cases prevented, or symptom-free days		12 (27)		[41,53,55,59-61,68,71-74,84]	
	Cost-minimization analysis	Used when 2 or more interventions or treatments are assumed to produce identical outcomes or equivalent effectiveness. Given that the outcomes are considered the same, the focus is entirely on minimizing costs.		5 (11)		[41,61,72,78,82]	
	Cost-utility analysis	Measures outcomes in terms of both quantity (life expectancy) and quality of life. It uses a metric called quality-adjusted life years or disability-adjusted life years to quantify health benefits		3 (7)		[41,61,72]	
**Technological safety and transparency**							
	Safety	Evaluation of an AI tool’s ability to avoid causing harm to patients by ensuring that it operates reliably, adheres to clinical standards, and mitigates potential risks		26 (59)		[42,47,49-56,60,62-68,71,73,75,78,80,81,83,84]	
	Transparency	Refers to the extent to which an AI tool’s processes, decision-making logic, and data sources are made understandable and accessible to stakeholders		27 (61)		[42,44,46,49,50,52-54,56,58-61,63-65,67,68,70,71,73-76,78,80,81]	
	Ethical oversight, human in command	Assesses whether the AI tool is designed to support human decision-making, allowing clinicians to maintain control and override AI decisions when necessary, ensuring AI complements rather than replaces human judgment		14 (32)		[41,42,45,49,51,64,65,67,68,70,74,75,78,80]	
**Scalability and impact**							
	Clinical effectiveness	Assesses how well the AI tool works in real-world practice, including its ability to achieve desired clinical outcomes across diverse populations and settings		26 (59)		[41-44,47-49,51-54,60,63-65,67-69,71-73,75,77,78,83,84]	
	Clinical efficiency	Focuses on the optimal use of resources (time, staff, and cost) to deliver care		8 (18)		[47,52,53,55,56,68,76,79]	
	Clinical utility	Refers to the practical benefits of a treatment or intervention in improving patient care, such as guiding clinical decision-making or reducing risks		14 (32)		[41-43,47,48,50,52,56,58,60,62,69,77,82]	
	Environmental impact	Evaluates how the development, deployment, and operation of AI tools affect environmental sustainability, such as energy consumption and carbon footprint		1 (2)		[75]	

^a^AI: artificial intelligence.

With our focus on assessing the long-term real-world impact of AI technologies in health care, we named the framework AI for IMPACTS. The criteria were organized into seven key clusters, each corresponding to a letter in the acronym: (1) I — integration, interoperability, and workflow; (2) M — monitoring, governance, and accountability; (3) P — performance and quality metrics; (4) A — acceptability, trust, and training; (5) C — cost and economic evaluation; (6) T — technological safety and transparency; and (7) S — scalability and impact.

## Discussion

### Principal Results

Through our systematic review of the literature, which culminated in the inclusion of 44 relevant papers, we conducted a narrative synthesis guided by the sociotechnical framework. This synthesis identified and categorized the key technical, social, and organizational criteria critical for the practical and effective implementation of AI technologies in health care. The results are organized into 7 main clusters, further divided into 28 specific subcriteria, providing a structured framework to address the multifaceted considerations highlighted in the reviewed literature.

By synthesizing and aggregating the assessment criteria from all included studies, we developed the AI for IMPACTS framework. This framework goes beyond focusing solely on technical metrics or methodological guidance at the study level. It integrates the clinical context and real-world implementation factors to ensure AI tools are evaluated holistically. Most criteria in our proposed framework can be aligned with existing frameworks, but none covers all relevant categories without extensions. For successful AI implementation in health care, it is essential to integrate these tools within the broader organizational context. Frameworks should account for the complexities of the sociotechnical environment, recognizing the interplay between technical, social, and organizational dimensions. Our consolidated framework achieves this by synthesizing and expanding existing frameworks for AI assessment in health care. It uses a sociotechnical approach to consider all contextual factors, their interactions, and the long-term real-world impact of these technologies in clinical practice.

The sociotechnical theory, which emphasizes the dynamic interplay between social, organizational, and technical aspects, provides a holistic approach to evaluating novel technologies [33]. This is critical in health care, where the successful implementation of novel technologies requires a balance of these factors to optimize both technology adoption and clinical outcomes [36]. Each component of the AI for IMPACTS framework reflects this sociotechnical foundation, as described below.

- I: integration, interoperability, and workflow — sociotechnical theory stresses the need for alignment between technology and workflow. This criteria cluster ensures that AI tools integrate seamlessly within existing systems and workflows, minimizing disruptions and supporting health care professionals in their work.
- M: monitoring, governance, and accountability — governance structures are vital for ensuring AI applications adhere to clinical standards and ethical norms. The sociotechnical theory supports the need for oversight that considers not just technical capabilities but also social and organizational responsibilities, promoting accountability in decision-making.
- P: performance and quality metrics — effective AI assessment requires robust performance metrics that span technical and clinical outcomes. By applying sociotechnical principles, this criteria cluster ensures that quality standards are met in ways that resonate with both technical requirements and patient care priorities.
- A: acceptability, trust, and training — for AI to be widely adopted, it must be trusted and understood by users. The sociotechnical theory emphasizes the role of social factors such as trust and user training, which are essential for fostering acceptance among health care providers and patients.
- C: cost and economic evaluation — costs are a key concern in health care. The sociotechnical approach underscores the importance of evaluating not just technical implementation costs but also the economic implications for patients and health care systems, ensuring that AI tools are financially sustainable and valuable.
- T: technological safety and transparency — safety and transparency are core to AI in health care, as they directly affect user trust and patient safety. The sociotechnical theory highlights that these technical attributes must be coupled with transparent communication and organizational processes that make AI’s functioning understandable and dependable.
- S: scalability and impact — sociotechnical principles stress adaptability within complex systems. This criteria cluster considers how AI can be scaled effectively across diverse health care settings, evaluating both technical scalability and the social and organizational impact for expansion.

By leveraging the sociotechnical theory, the AI for IMPACTS framework ensures that each criterion is evaluated in a way that respects the complex interdependencies between technical capabilities, social context, and organizational readiness, providing a balanced and comprehensive approach to AI assessment in health care. We selected the acronym IMPACTS to underscore our emphasis on real-world outcomes over isolated, study-level evaluations. This highlights our commitment to assessing the broader, practical effects in health care settings.

**Figure 3:**
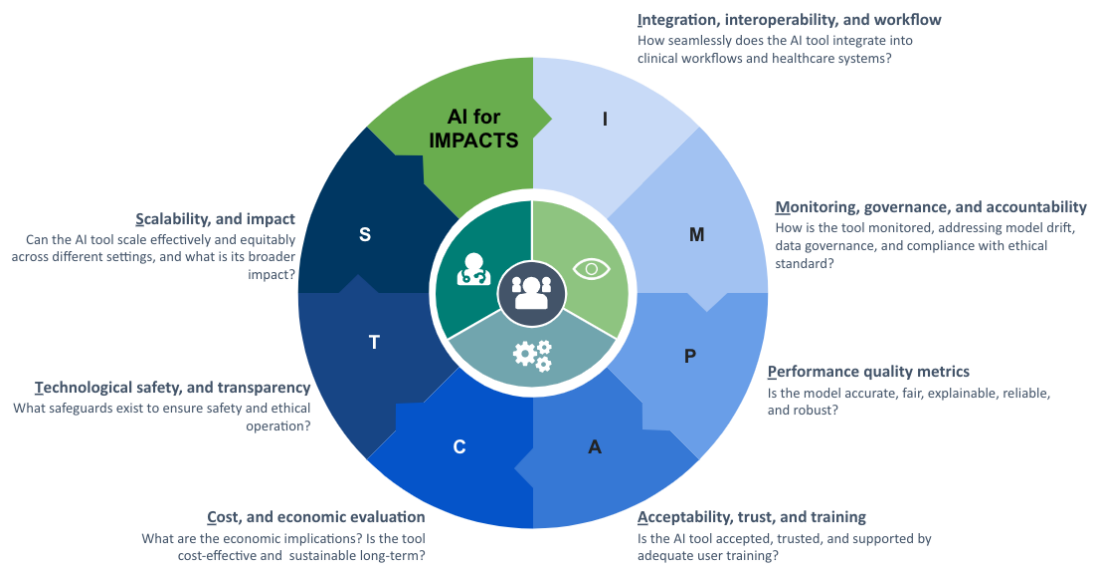
AI for IMPACTS: a comprehensive framework for evaluating the long-term real-world impacts of artificial intelligence (AI)–powered clinician tools.

Figure 3 depicts the 7 assessment clusters of the AI for IMPACTS framework. Each cluster contains multiple subcriteria, all of which are summarized in a comprehensive checklist presented in Table 3. The framework provides a systematic approach for evaluating AI’s holistic role and potential in health care applications. The following subsections provide a detailed analysis of each criteria cluster and their respective subcriteria, offering a comprehensive breakdown of how each factor contributes to the overall assessment.

**Table 3:** The AI for IMPACTS framework assessment criteria for evaluating the long-term real-world impacts of artificial intelligence–powered clinician tools.

Criteria			Assessment
**Integration**			
	Infrastructure	Does the deployment and scalability of the AI^a^ tool require additional technological, hardware, or software infrastructure beyond what is already available in the current clinical setting?	
	Interoperability	Does the AI tool seamlessly integrate and exchange data with various health care platforms and devices, ensuring interoperability across different systems without requiring significant modifications?	
	Workflow and organizational changes	Does the AI tool integrate smoothly into existing clinical workflows and health care operations, minimizing disruption while enhancing efficiency, communication, and the overall delivery of care?	
**Monitoring, governance, and accountability**			
	Accountability and liability	Is there clear attribution of responsibility for errors or outcomes, supported by well-defined legal and ethical frameworks that ensure accountability and proper recourse in the event of any issues?	
	Consent and data ownership	Does the AI tool have clear and robust processes for obtaining informed consent from patients, including transparent policies on data ownership, privacy, and control, ensuring patients fully understand how their data will be used?	
	Maintenance and updates	Does the AI tool have established processes for ongoing support, including regular updates and bug fixes, to ensure it remains effective, secure, and compliant with evolving medical standards and practices?	
	Monitoring and governance	Does the AI tool have systems in place for ongoing oversight of its performance, including regular assessments and audits to ensure ethical use, effectiveness, and adherence to relevant standards?	
	Regulatory compliance	Does the AI tool demonstrate adherence to established regulations throughout its entire life cycle, with systems in place for ongoing monitoring and reporting postdeployment to ensure continued safety, efficacy, and compliance with legal requirements?	
	Security and privacy	Does the AI tool have robust measures in place to protect sensitive patient data from unauthorized access and breaches, while ensuring full compliance with relevant privacy regulations?	
**Performance quality metrics**			
	Foundational metrics	These are application-specific metrics to ensure each tool is assessed appropriately based on its function: Diagnosis and prediction applications: use classification metrics (eg, accuracy, sensitivity, specificity, and area under the curve) for diagnosis tasks and regression metrics (eg, mean absolute error and root mean square error) for predicting continuous outcomes (classification, anomaly detection, and recommendation systems) Image and pattern analysis: focus on segmentation accuracy and reinforcement learning’s long-term performance optimization (eg, Dice coefficient, Jaccard index, and cumulative reward) Text and language processing applications: evaluate the accuracy and quality of AI-extracted or generated text (eg, completeness and relevance, empathy and engagement, floating-point operation count, and hallucination)	
	Explainability (ethics and trustworthiness)	Is the AI tool able to clearly show how it reached a specific decision or prediction in a way that clinicians can understand?	
	Interpretability	Is it easy for clinicians to understand the relationship between the input data and the AI tool’s outputs, without needing detailed technical explanations?	
	Fairness (equity)	Does the AI tool ensure fairness by avoiding systematic discrimination against any specific group, such as race, gender, or socioeconomic status, and promoting equitable outcomes in diagnoses and treatments?	
	Reliability, repeatability, and reproducibility (consistency and stability)	Does the AI tool demonstrate reliability, repeatability, and reproducibility by consistently delivering the same results over time, under similar conditions, and when applied to different data sets or used by different teams?	
	Robustness and generalizability (adaptability)	Does the AI tool demonstrate both robustness and generalizability by maintaining strong performance despite variations or noise in input data, and by performing well on new, unseen data from different hospitals or regions compared to its training data?	
**Acceptability, trust, and training**			
	Acceptance and adoption	Does the AI tool demonstrate strong acceptance by health care professionals and patients, including their willingness to adopt and integrate it into routine clinical practice?	
	Training and support	Does the AI tool provide comprehensive and readily available resources for users, ensuring they have the necessary guidance, training, and assistance to successfully implement and operate it in clinical practice?	
	Trust	Does the AI tool inspire trust among health care professionals and patients in terms of its reliability, accuracy, and ethical considerations, thereby positively influencing their willingness to use it?	
	Usability	Does the AI tool offer an intuitive and user-friendly interface that allows health care professionals and patients to interact with it easily and effectively, ensuring it enhances the user experience and integrates smoothly into clinical workflows?	
	User centricity (user, domain, and task type)	Does the AI tool effectively meet the specific needs, preferences, and contexts of its users, while addressing domain-specific requirements and supporting the relevant tasks for which it is intended?	
**Cost and economic evaluation**			
	Costs and economic evaluation	Does the AI tool provide financial value by enhancing care without imposing excessive costs on health care systems or patients, ensuring that its implementation is economically sustainable? This can be measured using one or more of the following methods*:* Does the AI tool demonstrate cost-effectiveness by offering a favorable balance between its costs and the health outcomes it achieves, such as life years saved, cases prevented, or symptom-free days, when compared to alternative interventions? Does the AI tool demonstrate cost-utility by providing measurable improvements in both life expectancy and quality of life, quantified through metrics such as quality-adjusted life years or disability-adjusted life years? Does the AI tool demonstrate cost-minimization by achieving equivalent outcomes or effectiveness compared to alternative interventions, while focusing on minimizing overall costs?	
**Technological safety and transparency**			
	Safety	Does the tool reliably adhere to clinical standards, consistently mitigate potential risks, and demonstrate the ability to avoid causing harm to patients through reliable operation and risk management?	
	Transparency	Does the AI tool provider ensure transparency by making its processes, decision-making logic, and data sources understandable and accessible to all relevant stakeholders?	
	Ethical oversight, human in command	Does the AI tool incorporate ethical oversight by ensuring that it supports human decision-making, allowing clinicians to maintain control and override AI-generated decisions, when necessary, thereby complementing rather than replacing human judgment?	
**Scalability and impact**			
	Clinical effectiveness	Does the AI tool demonstrate clinical effectiveness by consistently achieving the desired clinical outcomes in real-world practice, across diverse patient populations and health care settings?	
	Clinical efficiency	Does the AI tool demonstrate clinical efficiency by optimizing the use of resources, including time, staff, and costs, to effectively deliver care without compromising quality?	
	Clinical utility	Does the AI tool demonstrate clinical utility by offering practical benefits that improve patient care, such as guiding clinical decision-making or reducing risks during treatment?	
	Environmental impact	Does the AI tool minimize its environmental impact by considering sustainability in its development, deployment, and operation, including factors such as energy consumption and carbon footprint?	

^a^AI: artificial intelligence.

### Integration

This criteria cluster focuses on evaluating how effectively the AI tool integrates into existing clinical workflows and health care systems.

*Infrastructure* plays a crucial role in the successful implementation of AI tools in health care settings. Adequate computational power, specialized hardware, and robust IT infrastructure are often necessary to support the processing of large datasets and the operational demands of AI technologies [49,71]. This may include advanced components such as graphics processing units, which are not always standard in health care systems [55]. In addition, integrating these tools might require significant investment in new hardware or upgrades [60,69]. For cloud-based AI solutions, attention must be paid to network security and performance [55]. Ensuring infrastructure compatibility is essential for the smooth deployment and optimal functionality of AI in health care [41,54].

*Interoperability* ensures seamless integration with existing systems, such as electronic health records and imaging software. It allows AI tools to operate within current workflows without disrupting established clinical processes, enhancing data exchange across platforms [41,68]. It also ensures that AI tools adhere to industry standards, facilitating communication between different health care technologies and minimizing issues such as data misinterpretation or workflow inefficiencies [71]. Proper integration can reduce the resource burden on health care facilities and improve the overall usability and effectiveness of AI systems in diverse clinical settings [64].

Understanding the impact on *clinical workflows and organizational structures* is essential. AI tools must be seamlessly integrated into workflows to avoid disrupting clinical processes [49,82]. Evaluating how AI affects the redistribution of tasks among health care professionals and identifying necessary organizational changes are essential [64,67]. Poor integration or failure to align with clinical routines can negatively impact efficiency, increase cognitive burdens, and require significant resources to adapt systems [45,58].

### Monitoring, Governance, and Accountability

This criteria cluster focuses on evaluating how effectively the AI tool is monitored throughout its life cycle, addressing critical aspects such as model drift, data governance, and adherence to ethical standards.

Clarity on *accountability and liability* is essential when assessing AI tools in health care due to the potential risks involved in their implementation [49,81]. AI systems can make errors or offer recommendations that may not be followed by clinicians, raising complex questions about who is responsible when mistakes occur [54,78]. The lack of clear guidelines on whether liability lies with the developer, the health care institution, or the clinician using the tool poses significant legal and ethical concerns [55,84]. Proper assessment frameworks must ensure that accountability is well-defined, including clear roles for all stakeholders involved (eg, clinicians, developers, and institutions) particularly in cases of adverse events or errors [50,64,70].

*Data security, privacy, informed consent, and data ownership* are vital criteria for assessing AI tools in health care. These tools often require large amounts of sensitive patient data, which must be protected from unauthorized access, breaches, or misuse [75,83]. Ensuring compliance with relevant regulations, such as General Data Protection Regulation or Health Insurance Portability and Accountability Act, is essential to safeguard patient privacy [55,60,71]. In addition, clear processes for obtaining informed consent are critical, ensuring that patients understand how their data will be used [68,81]. Proper data ownership policies must also be in place, ensuring transparency around who controls the data and how it can be accessed or shared [49,78]. These measures are crucial for building trust and ensuring ethical AI deployment in health care settings [54,68].

*Regulatory compliance and certification* are essential but insufficient assessment criteria for AI tools in health care [21]. Although regulatory bodies like the FDA in the United States and CE marking in the EU set minimum safety and efficacy standards, there are significant gaps between legal certification and real-world clinical validation, workflow integration, and ongoing use [21,45]. For instance, FDA clearance does not always assure users that an AI tool will meet their expectations for effective performance in all clinical settings, leading to skepticism among health care professionals [21,84]. Similarly, in the EU, AI tools with CE marking are often assumed to be clinically validated, but many lack sufficient validation for real-world clinical use, such as in dementia diagnosis via magnetic resonance imaging [45,83]. These gaps highlight the need for stronger regulatory frameworks and postmarket surveillance to ensure AI tools are not only certified but also thoroughly validated and integrated into health care workflows for effective and safe use [21,72,76].

*Monitoring and governance* mechanisms, including feedback loops, are critical for ensuring the continued safety, effectiveness, performance, and reliability of AI tools in health care [71]. It is essential that the responsibility for monitoring these tools is shared between the developer, regulator, and the health care organization deploying the tool [84]. Developers are responsible for ongoing performance evaluations, including regular updates to address issues such as data drift or algorithmic failure [48,74]. Regulators must ensure compliance with postmarket surveillance requirements and set clear guidelines for monitoring practices [60,84]. Health care organizations must implement local oversight systems, ensuring that the AI tool continues to meet clinical needs without causing disruption or harm [49,64,65,71,83]. By assigning responsibility to all 3 entities, health care systems can ensure comprehensive, multi-layered oversight that addresses technical, clinical, and regulatory concerns [84].

The *maintenance and updating* of AI tools are critical to ensuring their continued effectiveness and safety in health care [71]. Regular updates, including adjustments to algorithms and reference datasets, are essential to avoid performance degradation and ensure accurate results [53,71]. Without proper maintenance, different software versions could introduce biases or inconsistencies, which might affect clinical outcomes [45,76]. Establishing clear protocols for updates, including version control and procedures for managing software changes, ensures that AI tools remain reliable and aligned with current medical standards, safeguarding patient care [50].

### Performance Quality Metrics

This criteria cluster focuses on evaluating the performance and quality of the AI tool by assessing key metrics such as foundational performance metrics, fairness, explainability, reliability, and robustness.

*Foundational performance metrics* play a crucial role in assessing the effectiveness of AI tools. The systematic review revealed that 59% (26/44) of studies primarily focused on accuracy, sensitivity, and specificity as key metrics. However, it is essential to consider application-specific metrics when evaluating AI performance, as different AI tools require tailored measures depending on their intended use. For example, diagnosis and prediction tools encompass applications like classification (eg, disease diagnosis), regression (eg, predicting disease progression), anomaly detection, and recommendation systems. These tools can be assessed through metrics such as accuracy, sensitivity, specificity, and the area under the curve for classification tasks [41,43,69] and mean absolute error and root mean square error for regression tasks [46,81]. Image and pattern analysis covers tasks such as image segmentation and reinforcement learning, using metrics like the Dice coefficient and Jaccard index for segmentation accuracy [87,88], and cumulative reward for evaluating reinforcement learning performance [89]. On the other hand, text and language processing applications, such as natural language processing and large language models, are assessed using metrics like relevance, engagement, empathy, token limits, hallucination rates, memory efficiency, and floating-point operation count [57,62,73]. These metrics ensure the AI tool is properly evaluated based on its intended use and technology type.

*Explainability and interpretability* are essential for ensuring the ethical and trustworthy use of AI tools in health care. These criteria allow health care professionals to understand how AI models arrive at their conclusions, fostering trust in their recommendations [47,83]. Explainability helps to demystify the AI’s decision-making process, making it transparent and accessible to users [67,68]. This, in turn, improves adoption, as clinicians are more likely to trust and rely on AI tools that are interpretable [49,60]. Ultimately, clear explainability supports ethical deployment, reducing risks associated with “black box” systems [73,78].

*Fairness or equity* ensures that AI models provide unbiased, consistent performance across diverse demographic groups, including those defined by race, gender, age, or socioeconomic status [62,66,83]. This criterion addresses the risk of bias in training data, including sample size and representativeness, which can lead to unequal treatment or outcomes for underrepresented populations [42,43,71]. By focusing on fairness, AI tools can avoid perpetuating disparities and contribute to more equitable health care delivery for all patients [55,63,71].

*Reliability, repeatability, and reproducibility* ensure that the AI tool can produce consistent outputs when presented with similar inputs, is repeatable under identical conditions, and is reproducible in diverse environments, including different institutions or patient populations [52,55,56,64]. Maintaining consistency and stability is essential for the tool’s trustworthiness and its broader applicability in real-world health care scenarios [54,84].

*Robustness and generalizability* are essential criteria for assessing the adaptability of AI tools in health care [42,83]. Robustness ensures the tool can maintain high performance even when exposed to slight variations in input data or operational environments [70,83]. Generalizability, on the other hand, evaluates whether the AI tool can effectively perform across different populations, clinical settings, or geographic regions beyond the environment in which it was trained [48,83]. These criteria ensure that AI tools remain reliable and effective when scaled or applied to diverse health care contexts [49,54].

### Acceptability, Trust, and Training

This criteria cluster evaluates user-centric aspects of the AI tool, focusing on its acceptance, trustworthiness, and the adequacy of user training and support.

User *acceptance and adoption* are crucial for the successful implementation and translation of AI-powered health tools in real-life settings [56,65,82]. Key challenges include fostering trust and confidence among health care professionals, ensuring ease of use, and integrating these tools seamlessly into clinical workflows [77]. User acceptance depends significantly on the perceived benefits, transparency, and safety of the AI systems [47,49,52]. Moreover, ethical concerns, the potential for bias, and the need for comprehensive testing also impact adoption [67]. Clinicians are more likely to embrace these tools when they complement human expertise and are introduced with adequate training and support, ensuring they enhance patient outcomes without compromising safety [68]. User acceptance and adoption of technology are typically measured through surveys (eg, Technology Acceptance Model and Unified Theory of Acceptance and Use of Technology) assessing factors like perceived usefulness and ease of use, as well as use metrics such as adoption rates, frequency, and retention.

*Trust* is built through factors such as validation, transparency, safety, privacy, and interpretability of the AI tool [62]. Both health care professionals and patients must trust that the AI tool is reliable, safe, and effective in clinical practice [60,68]. Validating AI performance using local data is essential to build clinician confidence, while demonstrating that the tool adheres to rigorous standards helps address concerns about its real-world application [49,84]. Trust also influences adoption, making it vital for the successful implementation of AI tools in health care [82]. User trust in technology is commonly assessed through surveys and trust scales, such as the Technology Trust Index, which evaluate key dimensions like reliability, competence, transparency, and security. Behavioral metrics, including use patterns and reliance during critical tasks, offer additional insights into how trust manifests in practice.

*User centricity* emphasizes the need for a clear understanding of the intended users, domain, and specific tasks the AI tool is designed to support [42,58,71]. AI tools must be tailored to meet the unique requirements of their end users, whether clinicians, nurses, or patients, and address the particular medical conditions they aim to diagnose, monitor, or treat [42,56]. Clarity in defining the tool’s intended use, the health care domain it serves, and the tasks it performs ensures that it delivers meaningful value in its practical application [64,73].

*Usability* ensures that the tool is user-friendly and intuitive for both health care professionals and patients [49,53]. An AI tool’s ease of use and minimal training requirements are essential for successful adoption [47,53]. Usability also impacts user satisfaction, influencing acceptance and trust in the system [52,77]. Proper design should minimize cognitive load, provide relevant information in context, and allow customization by users [71]. Evaluating usability ensures that AI tools can be effectively deployed in real-world clinical environments, enhancing rather than hindering care delivery [55,56].

Adequate *training* ensures that clinicians and other end users can effectively use AI tools, minimizing user error and maximizing the tool’s potential to improve patient outcomes [55,65,71]. Training programs should cover how to interact with the AI interface, interpret its outputs, and understand the tool’s limitations [49,54]. Continuous education is also crucial, and end users should not only be trained on interpreting the algorithm’s output but also be made aware of the factors that can affect its performance [64]. Moreover, accessible and responsive technical *support* is necessary to address user concerns, provide ongoing assistance, and maintain confidence in the AI tool’s reliability and safety over time [52]. Without proper *training and support*, the integration of AI tools into clinical practice may face significant barriers, limiting their overall effectiveness [45,65].

### Cost and Economic Evaluation

This criteria cluster evaluates the economic implications of the AI tool to determine its financial viability and long-term sustainability.

*Economic evaluation and cost* considerations are crucial in assessing AI tools in health care. AI interventions must demonstrate not only clinical value but also health economic impact to ensure their long-term sustainability [76,84]. This includes evaluating both direct costs, such as acquisition, maintenance, and implementation, as well as indirect costs like staff training or workflow disruptions [49,61]. Transparent and comprehensive economic evaluations help health care organizations determine the financial viability of AI tools, guiding decision-making on investments, reimbursement, and long-term sustainability [59,64,67,79]. Incomplete or unclear cost assessments can hinder AI adoption and create financial risks [72,79].

The choice of an economic evaluation method for an AI tool in health care depends on its intended use and desired outcomes. *Cost-effectiveness* analysis is useful when comparing costs with health outcomes like life years saved [41,59,68,71]. *Cost-utility* analysis is ideal when focusing on both life expectancy and quality of life improvements, measured in quality-adjusted life years or disability-adjusted life years [41,61,72]. *Cost-minimization* analysis is appropriate when the AI tool achieves similar outcomes as alternatives but aims to reduce costs [61,72,78]. The method chosen should align with the tool’s specific goals and intended health care impact.

### Technological Safety and Transparency

This criteria cluster focuses on evaluating the technological safety and transparency of the AI tool by assessing the safeguards in place to ensure safe and ethical operation.

*Safety* ensures that AI systems operate reliably and securely in clinical environments beyond laboratory settings and clinical trials [73,78]. This includes compliance with safety regulations, minimizing the risks of harmful outcomes, and maintaining high standards for long-term safety and patient protection [53,67,68]. Safety also encompasses the reliability of the AI model after its implementation, ensuring it consistently avoids errors and unintended consequences [47,65,78]. Ongoing monitoring, risk management, and thorough clinical validation are necessary to ensure that AI tools remain safe and effective in diverse health care settings and the long-term safety of constant updates [49,67,68,83].

*Transparency* is a critical assessment criterion for AI tools in health care, ensuring clarity in data processing, coding standards, and the overall functioning of AI systems [71,78,80]. Transparent models allow health care professionals to understand how decisions are made, promoting trust and enabling accurate assessments of the AI’s performance [63,67,68,73]. Clear documentation and disclosure of data processing methods, coding protocols, and the AI’s decision-making processes ensures accountability and reproducibility [42,54,64]. A recent review of 692 FDA-approved AI enabled medical devices highlighted major gaps in transparency and safety reporting [90]. Key data such as ethnicity (reported in only 3.6% of approvals), socioeconomic information (absent in 99.1%), and study participants’ age (missing in 81.6%) were often underreported [90]. In addition, only 46.1% of devices provided detailed performance results and only 1.9% were linked to scientific publications on safety and efficacy [90]. These findings underscore the urgent need for improved transparency and more comprehensive safety reporting to reduce algorithmic bias and ensure equitable health care outcomes.

*Ethical oversight and human in command* ensure human control and responsibility in the AI decision-making processes [64,70]. This criterion emphasizes that humans must retain ultimate authority over AI-generated decisions, particularly in critical health care scenarios [68,70]. Human in command ensures that clinicians can review, intervene, or override AI decisions, maintaining ethical standards and safeguarding patient outcomes [42,70]. This oversight protects against overreliance on automated systems and ensures that AI tools support, rather than replace, human judgment in clinical practice [45,68,80].

### Scalability and Impact

This criteria cluster focuses on evaluating scalability and impact by determining the AI tool’s clinical utility and effectiveness and examining its broader impact.

*Clinical effectiveness* focuses on the tool’s ability to positively impact patient outcomes [68,71,78]. This involves evaluating whether the AI tool contributes to better therapeutic results or patient-reported outcomes [43,71]. The assessment examines how well the AI tool integrates into real-world clinical settings and measures its tangible benefits in terms of patient health and health care quality [49,69]. Clinical effectiveness ensures that AI tools do more than function technically; they must provide meaningful improvements in patient care [47,51].

*Clinical utility* focuses on how effectively the tool supports clinical tasks and decision-making, including its ability to assist with diagnoses, treatment recommendations, and overall health care delivery [51,82]. Ensuring clinical utility means the AI tool must provide tangible benefits that align with clinical needs and enhance health care practices [43,79]. *Clinical efficiency* focuses on the tool’s ability to optimize resource use while maintaining or improving care quality [68]. This includes evaluating how well it improves productivity, reduces time spent on routine tasks, and streamlines workflows for health care professionals [47,53,55].

*Environmental impact* is an important, yet often overlooked, criterion for assessing AI tools in health care; only 1 out of 44 studies addressed this criterion. The energy consumption and resource use associated with developing, deploying, and maintaining AI systems, such as data centers, computational power, and device infrastructures, can lead to significant environmental harm, including e-waste and greenhouse gas emissions [75]. Implementing eco-responsible practices, such as energy-efficient computing and sustainable data storage, is essential to minimizing the ecologic footprint of AI tools [75].

### Practical Implications and Persisting Challenges

The wide array of frameworks and initiatives focused on AI assessment in health care shown in this systematic review highlights the significant lack of standardization in this field, creating additional challenges for stakeholders [43,70,71]. Faced with a growing number of assessment tools, they often struggle to determine which approach is most appropriate or how to apply it effectively [63]. This diversity in assessment methods can lead to confusion and hinder comparability [43,68,69,79]. Variations in data collection and evaluation methods, ranging from self-reported to objective measures, and from qualitative to quantitative assessments, only add to the complexity, further complicating the establishment of clear, universal guidelines for AI evaluation in health care [62].

Most frameworks included in this analysis were driven by the recognition that many existing methods for assessing AI tools in health care were not specifically tailored to AI-based medical devices or health care applications [62,65,67]. Traditional technology assessments often lack a critical focus on the unique, dynamic challenges and opportunities AI presents [57]. This underscores the need for health care–specific frameworks that account for the evolving nature and complexities of AI systems in clinical environments [60]. Moreover, existing frameworks tend to prioritize technical metrics such as algorithm accuracy, precision, and validation [62,73]. While these factors are undeniably important, this narrow focus often overlooks broader considerations, including clinical relevance, practical application, and long-term impact on patient outcomes [41,56,82]. Consequently, these frameworks can fall short in delivering a holistic evaluation of AI tools, which is essential for ensuring their safe, effective, and seamless integration into real-world health care settings [47,83].

This study builds upon and advances the ongoing discussion on AI assessment in health care, aiming to address the recognized gaps by developing the AI for IMPACTS framework. This proposed framework integrates technical, social, and organizational dimensions, ensuring that the adaptive nature of AI and the complexity of the health care ecosystem are fully considered. By encompassing these critical aspects, the framework provides a more comprehensive and nuanced approach to evaluating AI tools, helping shape the field and offering a robust method for assessing AI’s real-world impact in health care settings.

However, numerous challenges still remain. These challenges extend beyond just setting the assessment criteria, to include practical difficulties in implementing, validating, and standardizing these criteria across diverse health care environments. A key challenge in assessing AI tools in health care is the variation across different contexts and settings [64,71]. Most available evidence focuses on high-income countries, limiting the generalizability of findings to diverse health care environments, particularly in low- and middle-income countries [43,49]. Recent studies underscore the importance of collaborative efforts and context-sensitive solutions to effectively address the unique health care challenges faced in these regions [91]. Another challenge is the need for a multidisciplinary team of assessors. Effective evaluation requires collaboration among professionals from various fields, such as medicine, IT, and social sciences to ensure a comprehensive assessment [77,83]. This diversity of expertise is necessary to address the complexities of AI, from technical and ethical considerations to clinical relevance and real-world impact [55,65,84].

It is crucial to emphasize the importance of adequate training in assessment methods [69,74]. Many assessors may lack the specific expertise required to thoroughly evaluate AI-based tools [46]. Proper training in the complexities of AI technology and appropriate evaluation techniques is essential for conducting accurate and meaningful assessments [55]. Without this, the assessment process may be compromised, potentially leading to inaccurate or incomplete evaluations of an AI tool’s safety and effectiveness, which could undermine its implementation in health care settings [68]. Furthermore, the rapid pace of AI development, with AI-based medical devices having shorter product life cycles compared to traditional medical devices, underscores the need for more adaptive and fast-tracked health technology assessment processes [49,68]. Conventional health technology assessments are often too time-consuming, taking about a year to complete, which is incompatible with the fast-evolving nature of AI technologies [59]. Balancing the need for robust evidence with the dynamic nature of AI development is essential to ensure timely, informed decision-making and avoid delays in implementation and potential reimbursement [59,68].

### Limitations and Future Research

This study enhances the understanding of various criteria for assessing the quality and impact of AI tools in health care, but several limitations must be acknowledged. Relevant studies may have been missed due to language restrictions or limited database searches, and the exclusion of gray literature may have omitted valuable insights. In addition, no follow-up was conducted with the study authors to validate the findings, and manual reference searches were avoided to minimize citation bias. As a result, some relevant frameworks or assessment criteria may not have been captured in this review. Future research could expand to include studies in other languages, offering a more comprehensive understanding of potential interregional or intercultural differences in the assessment of AI tools in health care.

The critical appraisal of the frameworks included in this review highlighted that many papers discussing AI tool assessment in health care lacked rigorous validation, with some omitting the methods section entirely. To address this gap, we propose rigorously validating the AI for IMPACTS framework proposed in this work through a Delphi process. The Delphi method was selected as a means to validate the framework as it is specifically designed to achieve reliable expert consensus, particularly in addressing complex issues [92,93]. This method is widely recognized across various fields of medicine, especially for developing best practice guidance and clinical guidelines, where expert agreement is critical [94,95]. This approach will involve key stakeholders to critically apply, reflect on, and refine the framework, ensuring it is relevant, comprehensive, and user-friendly. The goal is to cocreate practical, accessible tools with industry experts that can support the effective evaluation of AI tools in real-world health care settings.

It is also important to highlight that new frameworks were published after the cutoff date of this systematic review, including the Organizational Perspective Checklist for Artificial Intelligence Adoption [96], Stanford’s framework for evaluating Fair, Useful, and Reliable Artificial Intelligence Models in Health Care Systems [97], and the Transparent Reporting of Ethics for Generative Artificial Intelligence checklist [98]. While an initial review shows that their assessment dimensions align with this work, a deeper integration will be undertaken before the validation study. This will ensure that the foundation for the Delphi process is as comprehensive and up-to-date as possible.

### Conclusions

AI has the potential to transform health care by improving clinical outcomes and operational efficiency. However, its adoption has progressed more slowly than anticipated, partly due to the absence of robust and comprehensive evaluation frameworks. Existing frameworks often focus too narrowly on technical metrics, such as accuracy and validation, neglecting real-world factors like clinical impact, workflow integration, and economic viability. Furthermore, the variety of frameworks and initiatives focused on AI assessment in health care, as highlighted in this systematic review, underscores a significant lack of standardization in the field, creating additional challenges for stakeholders and making it difficult to compare and implement AI tools effectively.

This study builds on and advances the ongoing discussion surrounding AI assessment in health care by developing the AI for IMPACTS framework. It aims to address key gaps identified in existing evaluation approaches, offering a comprehensive model that incorporates technical, social, and organizational dimensions. It is organized around 7 key criteria clusters: I—integration, interoperability, and workflow; M—monitoring, governance, and accountability; P—performance and quality metrics; A—acceptability, trust, and training; C—cost and economic evaluation; T—technological safety and transparency; S—scalability and impact.

While the framework provides a more holistic approach, significant challenges persist. The diverse contexts and settings in health care make it difficult to apply a one-size-fits-all framework. Multidisciplinary teams are necessary to evaluate AI tools thoroughly, as expertise from fields such as medicine, IT, and social sciences is required to address the complexities of AI. In addition, many assessors lack the specific training needed to evaluate these tools accurately. The rapid pace of AI development further complicates the assessment process, as conventional evaluation methods are often too slow to keep up with AI’s short product life cycles. To ensure successful AI integration in health care, adaptive and fast-tracked assessment processes are essential, allowing for timely decision-making and implementation while maintaining the necessary rigor.

## Data Availability

The data that support the findings of this study are available from the corresponding author upon reasonable request.

## Appendix

Multimedia Appendix 1 Critical Appraisal Skills Program appraisal of the included studies.

Multimedia Appendix 2 PRISMA (Preferred Reporting Items for Systematic Reviews and Meta-Analyses) checklist.

## Abbreviations

AI: artificial intelligence
CASP: Critical Appraisal Skills Program
CONSORT-AI: Consolidated Standards of Reporting Trials–Artificial Intelligence
EU: European Union
PICO: participants, intervention, comparators, and outcomes
PRISMA: Preferred Reporting Items for Systematic reviews and Meta-Analyses
