# Supplementary material for: AI for IMPACTS Framework for Evaluating the Long-Term Real-World Impacts of AI-Powered Clinician Tools: Systematic Review and Narrative Synthesis
Source: J Med Internet Res. 2025 Feb 5;27:e67485. doi: 10.2196/67485 (PMC11840377; doi:10.2196/67485)
Supplement: Multimedia Appendix 2 [file jmir_v27i1e67485_app2.docx]

| **Section and Topic** | **Item #** | **Checklist item** | **Location where item is reported** |
| --- | --- | --- | --- |
| **TITLE** | | |  |
| Title | 1 | Identify the report as a systematic review. | Title |
| **ABSTRACT** | | |  |
| Abstract | 2 | See the PRISMA 2020 for Abstracts checklist. | Abstract |
| **INTRODUCTION** | | |  |
| Rationale | 3 | Describe the rationale for the review in the context of existing knowledge. | Background and Objectives sub-sections under Introduction |
| Objectives | 4 | Provide an explicit statement of the objective(s) or question(s) the review addresses. | Objectives sub-section under Introduction  And the overview sub-section under Method |
| **METHODS** | | |  |
| Eligibility criteria | 5 | Specify the inclusion and exclusion criteria for the review and how studies were grouped for the syntheses. | Study selection sub-section under Method  And Table 1 |
| Information sources | 6 | Specify all databases, registers, websites, organisations, reference lists and other sources searched or consulted to identify studies. Specify the date when each source was last searched or consulted. | Search strategy sub-section under Method |
| Search strategy | 7 | Present the full search strategies for all databases, registers and websites, including any filters and limits used. | Search strategy sub-section under Method  And Textbox 1 |
| Selection process | 8 | Specify the methods used to decide whether a study met the inclusion criteria of the review, including how many reviewers screened each record and each report retrieved, whether they worked independently, and if applicable, details of automation tools used in the process. | Study selection sub-section under Method  And Table 1 |
| Data collection process | 9 | Specify the methods used to collect data from reports, including how many reviewers collected data from each report, whether they worked independently, any processes for obtaining or confirming data from study investigators, and if applicable, details of automation tools used in the process. | Data collection and Synthesis sub-section under Method |
| Data items | 10a | List and define all outcomes for which data were sought. Specify whether all results that were compatible with each outcome domain in each study were sought (e.g. for all measures, time points, analyses), and if not, the methods used to decide which results to collect. | Data collection and Synthesis sub-section under Method |
|  | 10b | List and define all other variables for which data were sought (e.g. participant and intervention characteristics, funding sources). Describe any assumptions made about any missing or unclear information. | Data collection and Synthesis sub-section under Method |
| Study risk of bias assessment | 11 | Specify the methods used to assess risk of bias in the included studies, including details of the tool(s) used, how many reviewers assessed each study and whether they worked independently, and if applicable, details of automation tools used in the process. | Critical appraisal sub-section under Results  And Multimedia Appendix 1 |
| Effect measures | 12 | Specify for each outcome the effect measure(s) (e.g. risk ratio, mean difference) used in the synthesis or presentation of results. | NA – this is a narrative synthesis (i.e. we used qualitative not quantitative analysis methods) |
| Synthesis methods | 13a | Describe the processes used to decide which studies were eligible for each synthesis (e.g. tabulating the study intervention characteristics and comparing against the planned groups for each synthesis (item #5)). | Data collection and Synthesis sub-section under Method |
|  | 13b | Describe any methods required to prepare the data for presentation or synthesis, such as handling of missing summary statistics, or data conversions. | NA – this is a narrative synthesis (i.e. we used qualitative not quantitative analysis methods) |
|  | 13c | Describe any methods used to tabulate or visually display results of individual studies and syntheses. | Data collection and Synthesis sub-section under Method |
|  | 13d | Describe any methods used to synthesize results and provide a rationale for the choice(s). If meta-analysis was performed, describe the model(s), method(s) to identify the presence and extent of statistical heterogeneity, and software package(s) used. | Data collection and Synthesis sub-section under Method |
|  | 13e | Describe any methods used to explore possible causes of heterogeneity among study results (e.g. subgroup analysis, meta-regression). | Critical appraisal sub-section under Results  And Multimedia Appendix 1 |
|  | 13f | Describe any sensitivity analyses conducted to assess robustness of the synthesized results. | NA – this is a narrative synthesis (i.e. we used qualitative not quantitative analysis methods) |
| Reporting bias assessment | 14 | Describe any methods used to assess risk of bias due to missing results in a synthesis (arising from reporting biases). | Critical appraisal sub-section under Results  And Multimedia Appendix 1 |
| Certainty assessment | 15 | Describe any methods used to assess certainty (or confidence) in the body of evidence for an outcome. | NA – this is a narrative synthesis (i.e. we used qualitative not quantitative analysis methods) |
| **RESULTS** | | |  |
| Study selection | 16a | Describe the results of the search and selection process, from the number of records identified in the search to the number of studies included in the review, ideally using a flow diagram. | Study Selection Flow and Characteristics of the Included Studies sub-section under results  And Figure 1  And Table 2 |
|  | 16b | Cite studies that might appear to meet the inclusion criteria, but which were excluded, and explain why they were excluded. | NA |
| Study characteristics | 17 | Cite each included study and present its characteristics. | Table 2 |
| Risk of bias in studies | 18 | Present assessments of risk of bias for each included study. | Critical appraisal sub-section under Results  And Multimedia Appendix 1 |
| Results of individual studies | 19 | For all outcomes, present, for each study: (a) summary statistics for each group (where appropriate) and (b) an effect estimate and its precision (e.g. confidence/credible interval), ideally using structured tables or plots. | Synthesized Assessment Criteria sub-section under results  And Figure 2  And Table 3  And Multimedia Appendix 1 includes an overview of each study and its results using a narrative synthesis rather than a quantitative approach |
| Results of syntheses | 20a | For each synthesis, briefly summarise the characteristics and risk of bias among contributing studies. | Critical appraisal sub-section under Results  And Multimedia Appendix 1 |
|  | 20b | Present results of all statistical syntheses conducted. If meta-analysis was done, present for each the summary estimate and its precision (e.g. confidence/credible interval) and measures of statistical heterogeneity. If comparing groups, describe the direction of the effect. | NA – this is a narrative synthesis (i.e. we used qualitative not quantitative analysis methods) |
|  | 20c | Present results of all investigations of possible causes of heterogeneity among study results. | Critical appraisal sub-section under Results |
|  | 20d | Present results of all sensitivity analyses conducted to assess the robustness of the synthesized results. | NA – this is a narrative synthesis (i.e. we used qualitative not quantitative analysis methods) |
| Reporting biases | 21 | Present assessments of risk of bias due to missing results (arising from reporting biases) for each synthesis assessed. | NA – this is a narrative synthesis (i.e. we used qualitative not quantitative analysis methods) |
| Certainty of evidence | 22 | Present assessments of certainty (or confidence) in the body of evidence for each outcome assessed. | NA – this is a narrative synthesis (i.e. we used qualitative not quantitative analysis methods) |
| **DISCUSSION** | | |  |
| Discussion | 23a | Provide a general interpretation of the results in the context of other evidence. | Discussion section |
|  | 23b | Discuss any limitations of the evidence included in the review. | Discussion section  And Critical appraisal sub-section under Results |
|  | 23c | Discuss any limitations of the review processes used. | Limitations and Future Research sub-section under Discussion |
|  | 23d | Discuss implications of the results for practice, policy, and future research. | Practical Implications and Persisting Challenges sub-section under Discussion |
| **OTHER INFORMATION** | | |  |
| Registration and protocol | 24a | Provide registration information for the review, including register name and registration number, or state that the review was not registered. | Overview sub-section under Method |
|  | 24b | Indicate where the review protocol can be accessed, or state that a protocol was not prepared. | Overview sub-section under Method |
|  | 24c | Describe and explain any amendments to information provided at registration or in the protocol. | Overview sub-section under Method |
| Support | 25 | Describe sources of financial or non-financial support for the review, and the role of the funders or sponsors in the review. | NA – we did not receive any funding for this work |
| Competing interests | 26 | Declare any competing interests of review authors. | Nothing to declare |
| Availability of data, code and other materials | 27 | Report which of the following are publicly available and where they can be found: template data collection forms; data extracted from included studies; data used for all analyses; analytic code; any other materials used in the review. | All data associated with this systematic review are available from the corresponding author upon reasonable request |

*From:*  Page MJ, McKenzie JE, Bossuyt PM, Boutron I, Hoffmann TC, Mulrow CD, et al. The PRISMA 2020 statement: an updated guideline for reporting systematic reviews. BMJ 2021;372:n71. doi: 10.1136/bmj.n71. This work is licensed under CC BY 4.0. To view a copy of this license, visit <https://creativecommons.org/licenses/by/4.0/>
